# Pazopanib-Induced Regression of Brain Metastasis After Whole Brain Palliative Radiotherapy in Metastatic Renal Cell Cancer Progressing on First-Line Sunitinib: A Case Report

**DOI:** 10.14740/wjon843w

**Published:** 2014-12-03

**Authors:** Mohan Hingorani, Sanjay Dixit, Anthony Maraveyas

**Affiliations:** a Department of Clinical Oncology, Castle Hill Hospital, Hull and East Yorkshire, NHS Trust, Cottingham, UK

**Keywords:** Renal cell cancer, Pazopanib, Brain metastasis

## Abstract

Pervious randomized studies have demonstrated survival benefit in favor of tyrosine kinase inhibitors (TKIs) compared to cytokines in metastatic clear cell renal cell carcinoma (RCC). However, the role of TKIs for treating brain metastasis from RCC remains unknown. Previous studies have reported possible activity of sunitinib and sorafenib in RCC patients with brain metastasis. We report on patient with metastatic RCC who responded to first-line sunitinib but then progressed with multiple brain metastasis, but with controlled extra-cranial metastatic disease. The patient was treated with whole-brain palliative radiotherapy followed by treatment schedule of pazopanib at standard dose of 800 mg/day which was associated with a response in brain metastasis. Subsequently, she was re-challenged at reduced dose of 600 mg/day and developed further response in metastatic brain lesions. She lived for more than 3 years from initial diagnosis of brain metastasis. This is the first case report of sequential TKI therapy for treating metastatic RCC with brain metastasis and supports the probable use of pazopanib as potent TKI for treating patients with cerebral metastasis.

## Introduction

The development of brain metastases has been reported in 10-25% of patients with renal cell carcinoma (RCC) with an average interval of approximately 17 months from original diagnosis and development of extra-cranial metastasis [1]. Treatment options include surgical resection, stereotactic radiosurgery (SRS), or whole-brain palliative radiotherapy (WBRT) depending on the nature (size and number) and location of metastasis. Surgical resection and SRS for small isolated lesions have been associated with good control and reduced rates of local relapse and 2- and 5-year survival rates of 30% and 12%, respectively [2-4]. In contrast, multiple brain metastasis has generally poor prognosis and WBRT has been associated with poor response with 1-year local control rate of 0-14% and median time to recurrence of less than 6 months [1, 2]. However, the prognosis of these patients may be changing in the current era of novel tyrosine kinase inhibitors (TKIs) that have shown promising activity in patients with brain metastasis.

We report on a case with metastatic RCC who developed response to first-line TKI therapy with sunitinib, but then progressed with development of multiple brain metastases. The patient was treated with WBRT and re-challenged with further TKI (pazopanib) that induced a partial response and regression of brain metastasis. The patient had unusually prolonged survival of 3 years from diagnosis of brain metastasis.

## Case Report

**Figure 1:**
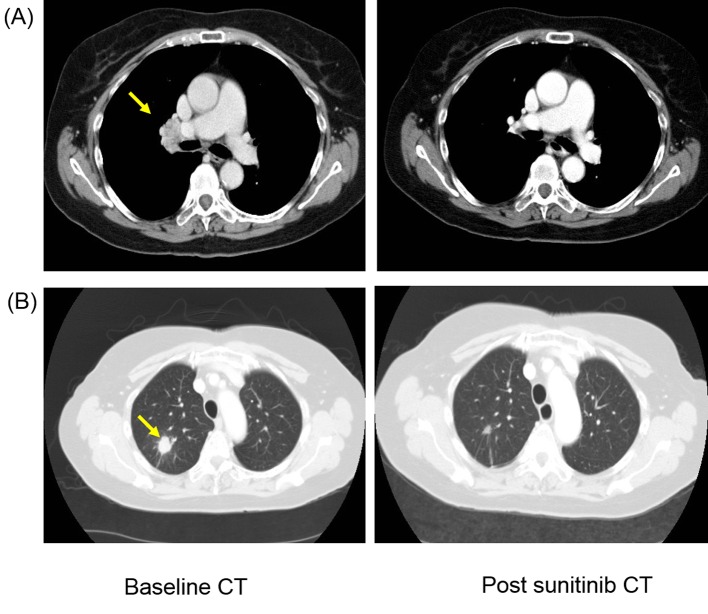
Patient developed response after first-line sunitinib therapy with complete resolution of (A) hilar lymphadenopathy (yellow arrow) and more than 50% reduction in size of (B) lung metastasis (yellow arrow).

A 73-year-old Caucasian female presented in January 2009 with a large 9 × 8 cm tumor involving the left kidney. She underwent a left radical nephrectomy and post-operative histology showed presence of typical clear cell carcinoma of kidney (Fuhrman grade 3) with involvement of renal vein with pathological staging of T3aN0 (TNM version-7) completely excised RCC. She did not receive any adjuvant therapy. She relapsed in February 2010 when a routine surveillance CT scan demonstrated metastatic lesion in the upper lobe of right lung with associated mediastinal and hilar lymphadenopathy. She was asymptomatic with WHO performance status of one and the hematological and biochemical profile was normal. She was classified as favorable risk based on the Memorian Sloan Kettering Cancer Center prognostic stratification model. She commenced TKI therapy sunitinib at dose of 50 mg/day based on 4 weeks “on” and 2 weeks “off” schedule. She underwent staging CT scan in September 2010 that demonstrated complete response in hilar lymphadenopathy and more than 50% reduction in size of lung metastasis (Fig. 1). She continued on sunitinib but presented in January 2011 with expressive dysphasia, right-sided weakness and generalized seizures and contrast-enhanced CT and MRI scan of brain demonstrated evidence of small multiple ring-enhancing lesions suggestive of multiple brain metastases. The staging CT showed no evidence of relapse outside the brain. At that stage sunitinib was discontinued and she was commenced on dexamethasone with improvement in neurological symptoms. Her case was discussed with neurosurgical colleagues who excluded any local therapy (surgery; SRS) in view of multiple nature of the lesion. Therefore, she was treated with WBRT using dose of 30 Gy in 10 fractions. She tolerated radiotherapy well but subsequently developed radiotherapy-related grade 3 tiredness and fatigue. Subsequently, she was managed with watchful expectancy and repeat imaging in April 2011 demonstrated no evidence of disease progression with stable appearances of brain metastasis.

**Figure 2:**
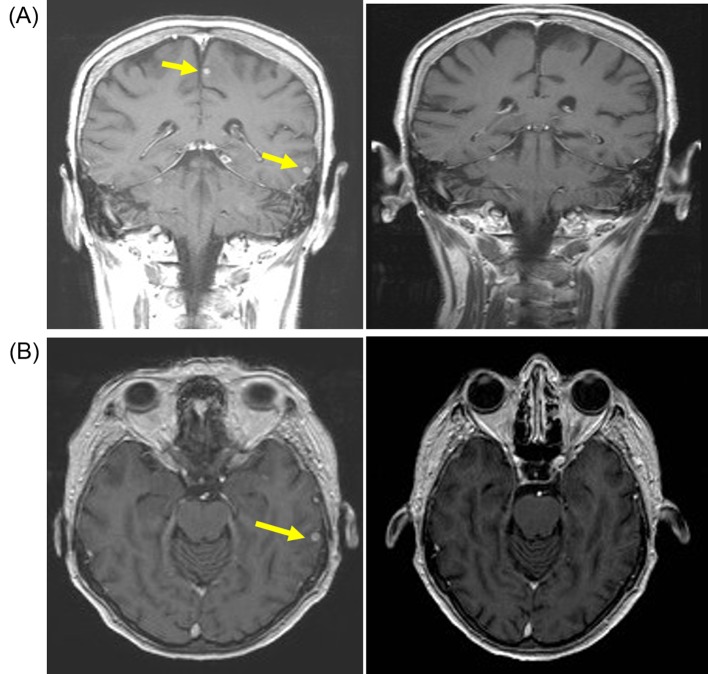
Patient developed response after pazopanib (800 mg/day) with resolution of brain metastasis. (A) Coronal T1-weighted scan with gadolinium contrast demonstrating small sub-centimeter contrast enhancing lesions (yellow arrow) on baseline MRI scan (left panel) that resolved after 3 months of pazopanib (right panel). (B) Axial T1-weighted baseline MRI scan with gadolinium showing small sub-centimeter metastasis (left panel) that resolved after 3 months of pazopanib (right panel).

**Figure 3:**
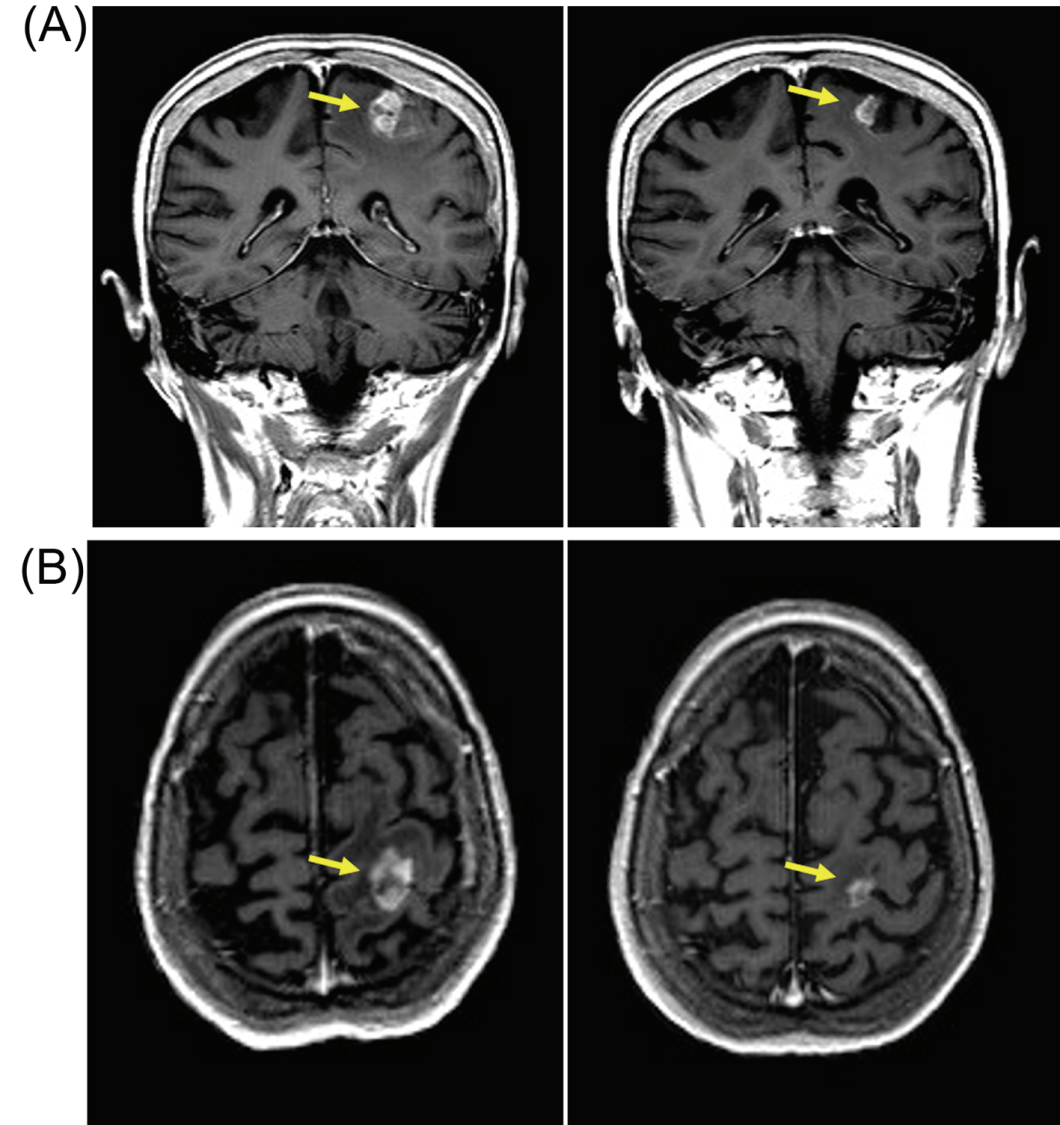
Patient developed response after re-challenge schedule of reduced-dose pazopanib (600 mg/day) with reduction in size of left parietal brain metastasis. (A) Coronal T1-weighted scan with gadolinium contrast demonstrating contrast enhancing lesions (yellow arrow) on baseline MRI scan (left panel) in left parietal region that resolved after 3 months of pazopanib (right panel). (B) Axial T1-weighted baseline MRI scan with gadolinium showing left parietal metastasis (left panel) that resolved after 3 months of pazopanib (right panel).

In June 2011 a follow-up CT showed small volume lung metastasis and stable appearances of brain metastasis. There was an improvement in her clinical condition and performance status (WHO grade 1-2), but she had commenced therapeutic anticoagulation with low-molecular weight heparin in view of below-knee deep vein thrombosis and pulmonary embolism. In view of reappearance of lung metastasis, she was commenced on pazopanib 800 mg/day that she tolerated well with no significant toxicities. She underwent repeat imaging 3 months later that showed an improvement in lung metastasis and also response in the brain metastasis (Fig. 2). She continued on pazopanib 800 mg/day, but the dose was reduced to 600 mg/day in December 2011 in view of symptoms of poor appetite and fatigue. Despite the reduction in dose of pazopanib, she continued to have poor tolerance of subsequent cycles and she embarked on treatment “holiday” with discontinuation of pazopanib in March 2012. She had progression-free interval of 11 months, but in February 2013 she developed progressive brain metastasis with enlarging lesion in the left parietal lobe. She recommenced pazopanib at 600 mg/day which she again tolerated poorly and the treatment was discontinued after 4 months in July 2013, but a repeat MRI scan confirmed response with reduction in size of brain metastasis (Fig. 3). She could not re-commence TKI therapy in view of deteriorating general condition, but repeat imaging in January 2014 showed no evidence of disease progression. She died in June 2014 and 40 months after her diagnosis with brain metastasis.

## Discussion

The management of RCC was revolutionized following the results from a pivotal phase III study published in 2007 demonstrating the effectiveness of TKI therapy with sunitinib improving survival outcomes compared to cytokines [5]. More recently, results from COMPARZ study demonstrated non-inferiority of pazopanib compared to sunitinib in first-line therapy of metastatic renal cancer [6]. Furthermore, pazopanib may have a better tolerance profile with reduced incidence of fatigue, hand-foot syndrome and oral toxicity with approximately two-thirds of patients preferring pazopanib compared to sunitinib in the double-blind patient-preference PISCES study [7].

Both sunitinib and pazopanib are small molecular TKIs with similar mechanisms of action and induce blockade of vascular endothelial growth factor (VEGF) types 1 through 3, platelet-derived growth factor (PDGF-α and PDGF-β), KIT, Fms-like tyrosine kinase-3 (FLT3), and colony-stimulating factor-1 pathways. There are pre-clinical data from animal studies that both sunitinib and pazopanib cross the blood-brain barrier (BBB) [8, 9]. In mice, concentration of sunitinib was higher in brain (7-fold) than in plasma, and in monkeys the brain concentration of sunitinib and its metabolite SU12662 were similar to the concentrations in plasma [8]. Previous phase II studies have demonstrated activity of pazopanib and sunitinib in patients with malignant gliomas [10], and for sunitinb in patients with brain metastasis from lung cancer [11].

There is limited evidence on the role of TKIs in the treatment of metastatic RCC with brain metastasis. All the pivotal phase III studies have excluded patients with brain metastasis. However, TKIs may have activity in treatment of brain metastasis as indicated by several case reports of response after exposure to sunitinib [12-15]. In an expanded-access series of 321 patients with brain metastases, the use of sunitinib was associated with an objective response in 26 of 213 evaluable patients (12%) [16]. Similarly, the use of sorafenib was associated with significant reduction in the incidence of development of brain metastasis (3% versus 12%) compared to placebo in separate analysis of 139 patients participating in the TARGET trial [17].

This case report is unique as the patient progressed on initial TKI therapy with sunitinib, but responded to further sequential TKI inhibition with pazopanib. There is some evidence on possibility of objective responses with sequential VEGF TKI inhibition [18, 19], but this has not been reported for pazopanib particularly in the context of synchronous brain metastasis. Our patient developed brain metastasis on sunitinib, but in the presence of complete clinical response in extra-cranial sites of metastatic disease. Indeed, another recent case report also highlighted the development of brain metastasis on sunitinib in the presence of complete response in the lungs, indicating that brain still represents a probable sanctuary site in these patients [20].

The clinical history of our case and the response in brain metastasis observed with pazopanib raises some extremely important and pertinent questions. Does the development of brain metastasis on TKI therapy in the presence of response in extra-cranial sites of metastatic disease be truly characterized as TKI failure, and what is the best therapeutic approach for these patients? It is more than likely that the development of brain metastasis in this situation represents inability of drug to cross the BBB, rather than representing loss of drug activity. Therefore, appropriate treatment of brain metastasis followed by re-challenge schedule of TKI appears to be a reasonable therapeutic paradigm to follow in these circumstances.

In our patient MRI showed multiple small brain metastasis and therefore patient received WBRT compared to surgery or SRS therapy. We decided to treat the patient with pazopanib given the favorable tolerance profile of the drug as the patient had experienced significant fatigue following completion of radiotherapy. It remains unknown whether the response to pazopanib may have been influenced by WBRT with its complex modulation of blood brain permeability, and whether re-challenging the patient with sunitinib may have induced a similar response. Does the response in brain metastasis induced by pazopanib in a patient progressing on sunitinib imply that pazopanib may be a more effective agent for treating brain metastasis? In contrast to sunitinib and sorafenib, there is no definite evidence on the possible activity of pazopanib in metastatic RCC with brain metastasis. We searched the literature and could identify only one report on pazopanib-induced response in brain metastasis in metastatic renal cancer. The report described a patient with papillary variant of RCC with multiple brain metastasis who was treated with WBRT followed by multiple lines of systemic therapy. Patient was treated with sunitinib, everolimus, standard-dose pazopanib (800 mg/day), sorafenib, and high-dose pazopanib (1 g/day). Patient was developing progressive brain lesions on standard-dose pazopanib which responded when the dose was escalated to 1 g/day. The patient had survival of 23 months from diagnosis of brain metastasis. The authors hypothesized that there may be a dose-response relationship with probable superior penetration of BBB with the increased dosage of pazopanib that reinforces the principles and importance of drug titration with targeted therapy [21]. In contrast, our patient responded to standard (800 mg/day) and reduced dose of pazopanib (600 mg/day) that continued to show sustained activity in two different schedules of treatment. Furthermore, there was durable progression-free interval after each schedule of treatment. There were no significant toxicities observed from pazopanib in relation to brain metastasis, particularly there was no evidence of hemorrhagic complication despite the concomitant use of therapeutic anticoagulation. The drug was discontinued in view of deteriorating general condition and performance status rather than progression of brain metastasis. Indeed, the patient had an unusual prolonged survival of 40 months from diagnosis of brain metastasis.

In summary, this is the first case report of activity of pazopanib in brain metastasis arising from conventional renal clear-cell carcinoma. This report demonstrates robust and sustained activity of pazopanib for treating brain metastasis in metastatic RCC arising on background of progression on first-line sunitinib. This report highlights that sequential TKI inhibition may be an appropriate therapeutic strategy to adopt in patient with brain metastasis. There should be further research in the role of pazopanib for treating brain metastasis in metastatic renal cancer.
